# Towards standardized mechanical characterization of microbial biofilms: analysis and critical review

**DOI:** 10.1038/s41522-018-0062-5

**Published:** 2018-08-20

**Authors:** Héloïse Boudarel, Jean-Denis Mathias, Benoît Blaysat, Michel Grédiac

**Affiliations:** 1 Université Clermont Auvergne, CNRS, SIGMA Clermont, Institut Pascal, F-63000 Clermont-Ferrand, France; 2 Irstea, UR LISC Laboratoire d’ingénierie des systèmes complexes, 9 avenue Blaise Pascal, CS 20085, 63178 Aubière, France

## Abstract

Developing reliable anti-biofilm strategies or efficient biofilm-based bioprocesses strongly depends on having a clear understanding of the mechanisms underlying biofilm development, and knowledge of the relevant mechanical parameters describing microbial biofilm behavior. Many varied mechanical testing methods are available to assess these parameters. The mechanical properties thus identified can then be used to compare protocols such as antibiotic screening. However, the lack of standardization in both mechanical testing and the associated identification methods for a given microbiological goal remains a blind spot in the biofilm community. The pursuit of standardization is problematic, as biofilms are living structures, i.e., both complex and dynamic. Here, we review the main available methods for characterizing the mechanical properties of biofilms through the lens of the relationship linking experimental testing to the identification of mechanical parameters. We propose guidelines for characterizing biofilms according to microbiological objectives that will help the reader choose an appropriate test and a relevant identification method for measuring any given mechanical parameter. The use of a common methodology for the mechanical characterization of biofilms will enable reliable analysis and comparison of microbiological protocols needed for improvement of engineering process and screening.

## Introduction

Biofilms are commonly defined as complex systems, comprising consortia of bacteria lodged in a three-dimensional extracellular matrix. Unlike the planktonic lifestyle, biofilms offer bacteria, protection against environmental, chemical, and mechanical stresses.^1^ The matrix fills the space between bacteria and produces a mechanical cohesive stability. Thanks to this stability, microorganisms embedded in biofilms possess a high survival and persistence potential. Biofilms are accordingly ubiquitous, and will colonize all surfaces in non-sterile environments that offer sufficient humidity for microbial life. Evidence has gradually accrued showing that the proportion of bacteria living in biofilms is largely predominant over planktonic bacteria. Recently, the biofilm has been considered as the default bacterial lifestyle, and it is thought that planktonic single cells may only be a transitional lifestyle of bacteria.^2^ Over the past decades, the microbiology community has taken a growing interest in biofilms,^3^ and the importance of biofilm life is now universally recognized. Biofilms are considered as a contamination vector because the detachment of biofilm bulk fragments under flow conditions facilitates bacteria dissemination and thus contamination. They remain therefore a potential cause of serious and persistent problems.^4^ Particularly, the biofilm lifestyle promotes bacterial colonization in medical devices, and more generally in all biomaterials,^5^ enhancing nosocomial infections^6^ or worsening pathological conditions.^7^ Nevertheless, biofilms are progressively intentionally engineered in biotechnological and bioengineering process. Indeed, diverse ecological processes exploit biofilm stability, particularly in bioremediation, wastewater treatment,^8^ and biofuels or nanomaterials synthesis.^9^ Characterizing biofilms is thus a broad concern with far-reaching societal implications. Dealing with the socioeconomic implications of biofilms thus requires a full understanding of the mechanisms underlying their persistence, dissemination, and transmission. A deeper understanding of mechanical behavior of biofilms is required to help eradicate or control harmful biofilms as it measures how bacteria bind together and biofilm dissociates. For instance, enhanced knowledge and advanced modeling of the mechanical properties of biofilms are crucial to understand the physical stability of biofilms, help improve cleaning procedures (intensity of the mechanical load of jet), help optimize operational parameters (fluid flow in water distribution pipelines) or help develop therapeutics strategies. In this context, many research groups have been working on these structured ecosystems.^10^ Since the early 2000s biofilm mechanics has emerged as a research theme. This aspect has been extensively reviewed in recent studies.^11–16^ The biofilm field attracts the attention of transversal communities and is getting abundant. Studying biofilms is challenging owing to their inherent properties: structurally they are highly heterogeneous and complex; in addition, bacteria are living systems and biofilms evolve rapidly. Intra-sample and sample-to-sample variability in results are thus to be expected. Furthermore, many different mechanical testing methods are used in the community, and literature values often differ by several orders of magnitude^14^ for the same bacterial strain. In the following, “bacterial strain” will refer to the kind of bacteria (*Pseudomonas aeruginosa*, *Streptococcus mutans*, …) whereas “strain” alone will refer to the mechanical term meaning the deformation of the biofilm matter under an applied load. It is a recurrent discovery that results are highly method-dependent. Difficulties in implementing these numerous tests and interpreting their results can lead to misleading conclusions. Yet the identified mechanical properties are then used to compare protocols such as antibiotic screening. Nowadays, the research community aims at introducing standards to manage and to analyze biofilms data in order to facilitate their comparison. Efforts were already being made to homogenize the process across the biofilm field. For instance, two platforms, MIABiE^17^ (Minimum Information About a BIofilm Experiment) and BiofOmics^18^ have been implemented to provide first public guidelines about the minimum information required that must be documented and stored, and then the database which collects biofilm experiments data on a systematic and standardized basis. Nevertheless, the mechanical interpretation of results has seldom been correctly addressed or emphasized in the literature. Here we review the issues and difficulties of the mechanics of biofilms. Beyond an update of the technical aspects of existing methods available for characterizing biofilm materials, our main purpose is to question microbiologists on the relevance of the available parameters, on the way to perform mechanical tests and on the necessity to share a unified terminology and protocols in the biofilm community. Finally we offer the reader guidelines that can be used as a support for deciding which is the best method for identifying mechanical properties and to choose which of these properties is the most relevant. Concerning the relevance of these properties, the aim is not to provide standards but rather common bases and good practices for the evaluation of mechanical parameters. Indeed, the issue of standardization is too fetched. This is the main task of standard-setting body.

## Biofilms mechanics: a microbiological significance

Mechanical parameters are an appealing outcome for microbiologist and the role of mechanics should not be underestimated. The mechanics of microbial biofilms is a cross-disciplinary theme that brings together concepts of engineering mechanics, chemical, physico-chemical, biochemical, and microbiological knowledge. Here we present the microbial objectives that should require a mechanical approach.

### Understanding the biofilm life-cycle

#### Impact of environmental stimuli on biofilm formation

The matrix accounts for up to 90% of the dry mass of biofilms and is linked to many functionnalities within the biofilms life-cycle.^19,20^ A fuller understanding of the mechanical properties of biofilms would help elucidate the interplay between the molecular mechanisms that govern the life cycle of a biofilm,^21^ from adhesion to dispersal.^22^ In their natural environment, biofilms are subject to external loads as fluid flow. It has been shown that bacterial mechanosensing gives rise to an active response to mechanical stress.^11^ Biological responses are influenced by changes in biofilm physical integrity. Hence the mechanical characterization of biofilms would definitively provide a better understanding of biofilm formation, and more specifically of the roles the different protagonists play in the microbial community.^23^ Mechanics influences differentiation of bacteria^24^ and similarly, bacterial differentiation spawns various biofilm physical features. The link between bacterial mechanosensing and changes on biofilm mechanical properties has to be further explored but it already exists evidences on the relationship between the two mechanisms. Shear stress has been shown to infer on biofilm structure for *Bacillus cereus*^25^ and *Pseudomonas fluorescens*.^26^ Shear stress affects production of the exopolysaccharides which constitutes the biofilm matrix such as Pel or Psl in the biofilm of *Pseudomonas aeruginosa*.^27^ Stress has also been shown to increase cyclic di-GMP level^28^ which is directly linked to the type IV pili expression, governing the transition from planktonic form to biofilm lifestyle^29^ for *Pseudomonas aeruginosa*. Growth and competition for nutrients leads to different patterns of cells colony in *Pseudomonas aeruginosa*.^30,31^ Studying mechanical properties could explain the mechanisms promoting distinct colonization shapes. The differentiation of bacteria tends to favor biofilm survival potential, and enhances its mechanical strength. The mechanical behavior is partly a function of bacterial gene product and studies related to operating conditions show that chemical stimuli (pH, nutrients, surfactants, etc.) and mechanical properties are closely interdependent.^32^

#### Viscoelastic behavior

Until now the microbiological community has agreed that biofilms are viscoelastic materials.^33–35^ In other words, biofilms are able to dissipate energy coming from external forces and to withstand external mechanical stress.^36^ The mechanics of biofilms is an important factor in determining how biofilms break up, disperse, and seed new biofilms under mechanical perturbation, such as flow.^37^ Mechanical properties of the biofilm can explain the trends towards the biofilm either spreads and colonizes new surface or strengthens. Especially, an important issue in health sector is the formation of flexible three-dimensional filaments called streamers,^38–40^ which give rise in clogging pipes in medical devices. This phenomenon is highly linked to the mechanical properties of EPS matrix,^41^ especially the viscoelasticity.

#### Antimicrobial screening

Mechanics of biofilm also deals with the screening of antibiofilm molecules. Prophylaxis and treatments are seldom issued with considering the possibility of a biofilm-related infection and most of antibiotics dedicated to planktonic bacteria often fail.^42^ One reason for the inefficacy of current treatments is the increased tolerance or resistance of biofilms to antibiotics.^43^ The poor diffusion of drugs in the biofilm, and the transformation of cells into persisters^4^ favor the recalcitrance of biofilms. Finding active antibiofilm substances and their corresponding efficacious doses is a major goal of current research in microbiology.^42,44^ Biofilm resistance to antibiotics is highly dependent on the structure of the EPS matrix. One possible strategy is to target the extracellular polymeric substances with matrix-degrading enzymes or with quorum-sensing inhibitors.^42^ Change of mechanical properties^32,45,46^ of biofilm upon antibiotic treatment can be a mediator to determine the operating mode and efficiency of an antibiotic.^47^ These parameters would act as biomarkers of biofilm-related infection progression or slowdown. Mechanical properties are used to determinate the impact of biocides on EPS matrix integrity. Several studies have observed the effect of biocides on the biofilm mechanical response.^48^ For instance, *Pseudomonas aeruginosa* and *Streptococcus epidermis* biofilms are modified by ciprofloxacin, glutaraldehyde, and urea.^46^ The study of the time course of mechanical properties before and after a treatment allows the screening of new entities able to inhibit or delay the formation of biofilms. Mechanical properties help determine the operating mode of an antibiotic, a biocide or any compound, e.g., whether it merely disperses the biofilm by impairing biofilm cohesion, or actually kills the bacteria. Moreover, one promising prospect is to combine chemical and mechanical strategies^42,47^ to target viscoelastic properties of bacterial biofilms. The chemical treatment reduces the cohesiveness^49^ or the stiffness^48^ of biofilms, and thus decreases the force needed to eradicate them or enhance the diffusion of biocides within the EPS matrix.^50^ The mechanical properties of biofilm could be used to probe quantitatively the biofilm cohesiveness, make clinical diagnoses, or adapt therapies to types of infection. Another need in chemical screening is the development of methods or devices to detect the presence of biofilms promptly before^51^ and after selected treatments, or simply to diagnose biofilm-associated infection stages.^52^

### Designing mechanical monitoring strategies

Both harmful and beneficial biofilms are challenged naturally by mechanical forces. In the first case, the design of mechanical cleaning strategies requires knowledge of biofilm strength. In the second case, a study of the mechanical properties of the biofilm is needed to sustain the reliability of the biofilm functions. Understanding what determines the strength of the biofilm is thus a major issue for microbiologists. Controlling the mechanical stability of biofilm offers one lever for monitoring their presence.

#### Mechanical removal of biofilms

Using biocides in antibiofilm strategies is sometimes inappropriate for biofilms outside the body or unsuccessful due to increasing resilience of biofilms to antibiotics and remaining biological substrates susceptible to be reused by bacteria.^53^ Repeated applications of cleaning cycles on industrial setup^54^ or on medical devices as endoscopes,^55^ tracheostomy tube^56^ have been shown to be a failure or to enhance the opposite effect and promote the resistance of bacteria. Mechanical force is another management approach in order to weaken and clear biofilms while bacteria show rising antibiotic resistance. Unlike the chemical strategy, mechanical removal is not a species-specific way to eliminate the biofilms. Biofilm management involves removing biofilms by imposing a flow or a mechanical load to overcome forces which keep the biofilm together and bonded to the surface. The detachment can be partial—cohesion loss—or complete—adhesion loss. The EPS matrix supports the mechanical stability of the biofilm through physico-chemical interactions. The aim is to disassemble EPS matrix or to detach biofilm clusters by weakening the cohesive forces. Measuring the ability of biofilms to withstand stress provides information and indicates future directions for the design of biofilm-cleaning tools. Mechanical studies measure parameters which enrich mechanical models and then improve the comprehension of the behavior of biofilms subjected to forces. Many studies have accordingly sought to determine at what point a biofilm fails or disperses when exposed to loading, in particular under the influence of hydrodynamics stress.^57^ In medical devices, surfaces are often cleaned using a pressurized water brush, in the same way that cariogenic biofilms are commonly removed mechanically with a toothbrush, but a more drastic method consists in generating a fluid stress in the form of high-velocity water droplets,^58,59^ microsprays, water jets, or air bubbles.^60^ Actually, fluid jet lavage is used to remove infected bacteria from tissue or abiotic surface. Moreover, it is now well known that the biofilm life-cycle includes dispersal^22,61^ of planktonic bacteria that enables biofilms to spread. The detachment of biofilm fragments driven by the flow can also lead to the transmission of pathogens to drinkable water.^62,63^ To limit contamination, microbiologists have to know precisely when detachment of biofilm fragments under flow stress occurs. Particularly interesting is then the study of biofilms behavior under fluid solicitation. The latter can cause biofilm to flow across surfaces in ripple and wrinkle structures. Another problem is the management of channel clogging by filamentous streamers.^64^ For instance, the tolerance of *Streptococcus mutans* to shear stress has been extensively studied to improve the mechanical removal of dental plaque.^65^ Reclamation and reuse is problematic in terms of microirrigation issues.^66^ Actually, biofilm formation provokes pipes clogging and industry has to process mechanical blockages. The use of adapted flow rate of water is based on the knowledge of the shear strength of the biofilm which cover pipes. Food and engineering industries need physical biofilm cleaning with strong shear flow.^67,68^ In addition to being reliable, the mechanical cleaning solutions should be eco-friendly.^66^ Furthermore, mechanics can be used as a mean to deliver more efficaciously antibiotics.^69^ Mechanical action is a possible way to increase the antibiotic susceptibility by enhancing chemical diffusion or the action of antibiotics^70^ which are more efficacious on planktonic bacteria.

#### Preventing biofilm formation

The most obvious strategy to forestall biofilm formation is to prevent the attachment of planktonic cells. Concerning biofilm prevention, the main antifouling strategies are surface coating^71^ and surface physico-chemical modification.^72^ Development of such surfaces is highly inspired of biomimetics.^73^ The principle is to offer an unfavorable substratum to avoid the bacterial adhesion and the biofilm formation. Understanding and quantifying biofilm adhesion capacities provides a way to compare surfaces and environmental living conditions, opening perspectives for designing hygienic surfaces to which bacteria cannot initially adhere and where a biofilm will thus not form. Mechanical properties can serve as quantitative criteria against which performance can be measured. Release of biocides or silver from surface coatings, micropillars^74,75^ or bioinspired nanostructural topography^76^ are some examples of strategies used in low adhesion surfaces. The control of topographical and physio-chemical properties of the substratum such as roughness, charge, tension, and hydrophobicity, stiffness^77^ may offer insights for developing surfaces that inhibit biofilm formation. The conditioning film, the layer formed on a solid surface by the adsorption of organic matter, can either enhance or reduce initial attachment of bacteria according to its composition.

#### Benefiting from biofilms in bioprocesses

Biofilm stability, availability and low cost make the biofilms the solution for many bioprocesses, in which they are used for these actions on deleterious organic and inorganic components^78^ or for these bio-electrochemical properties. Actually, due to the stress protection and adaptation provided by the matrix, bacteria living in biofilms are preferred over planktonic one in many bioprocesses. Bioremediation benefits from the metabolic reactions for the clean-up of environmental pollutants such as hydrocarbon contaminants or heavy metals using microorganisms.^79^ Biofilms are used in bioreactors for the production of desired compounds or for these applications in bio-nanotechnology.^9^ Benefiting biofilm formation and mechanical stability must be controlled as biofilms experience compressive, tensile or shear forces in real-world situations and these solicitations can damage beneficial biofilm.

Knowledge on biofilm mechanics is of particular interests for effective design and use of applications in which biofilms are either actively or passively involved. More specifically, parameters inferred from mechanical studies enrich mechanical models and help improve the comprehension of the behavior of biofilms subjected to environmental stress or user stimulus. For instance, knowing when the biofilms loss its rigidity is an appealing outcome to better deliver drugs in the case of cleaning procedures. Furthermore, cohesive forces can be sought using mechanical models to optimize operational parameters in physical removing strategies. Hence, developing an interdisciplinary understanding of bacterial biofilms behavior seems to be relevant to prevent their formation, treat biofilm-related infections, disrupt recalcitrant biofilms or harness the metabolic properties of biofilms.

## Measuring relevant mechanical parameters

The biofilmology community has to reach an agreement on the parameters at stakes for each of the microbiological issues. The antimicrobial screening mediated by biofilm mechanical properties, the biofilm release under the influence of an adapted flow rate of water in clogging pipes or the development of effective cleaning strategies are some of appealing issues of the mechanics of biofilms. Biofilm mechanics requires a common terminology to avoid fallacious analogies between results obtained following different routes. In the following part, we look at microbiological issues of interest in biofilms from a mechanical standpoint. The measurement of these properties following common bases is significant as it allows the determination of important tool as an antimicrobial screening index or an accurate shear stress needed to overcome clogging in water pipe. Depending on the microbiological issue discussed in the previous part, mechanical parameters can be used for several purposes: as indicators allowing to follow a change in the mechanical behavior or as a threshold value for monitoring the presence of a biofilm. A conceptual overview of the place of mechanics in the microbiological community often addressed in the literature is given in Table 1.

**Table 1:** Roles of mechanics in microbiological community

What are the microbiological needs?	Which place for mechanics?	Which mechanical parameter?	Applications
Knowledge about the biofilm life-cycle	Indicator of behavior change	Change in material properties before failure (elastic moduli, strain) Qualitative understanding (viscoelastic behavior)	Understanding the physical stability of biofilms Helping the development of therapeutics strategies (antibiotic screening) Understanding the streamer formation
Mechanical control strategies	Threshold value	Loss of cohesive or adhesive strength	Improvement of operational parameters in irrigation systems Improvement of biofouling management in hydrated environments and cleaning procedures (detaching biofilm clusters or disrupting extracellular matrix)

### Defining mechanical variables as microbiological proxy

Defining quantitative enough mechanical parameters to transpose physical properties into an indicator allows a monitoring of the change in the rheological behavior of the biofilm, the stiffness of the biofilm matrix and other physical features of the biofilm. Especially, the study of biofilm behavior without failure is interesting to understand mechanisms governing the biofilm life-cycle. A biofilm experiencing a mechanical perturbation changes its static equilibrium. Under external load, the equilibrium is broken, molecules constituting the biofilm are rearranged and polymeric chains of the matrix are modified. When loads are below a critical value, a biofilm sample will only deform without breaking. In mechanics, the parameters at stakes for the evaluation of the material properties are defined by the framework of continuum mechanics. Contrary to discrete approaches, continuum model treats the matter as a homogenous whole in terms of mechanical properties. It is a simplification where biofilm spatial heterogeneities are smoothed. Stress and strain experienced by a sample during a mechanical test are linked by a behavior law, introducing material parameters. Parameters probe the resistance to shape deformation or the deformability of the biofilm. These parameters are of great interest as quantitative probes to study mechanisms of biofilm growth and development. Indeed, it is interesting to conduct mechanical analysis according to bacterial gene expression or environmental conditions to reveal potential differences in mechanisms of biofilm formation.^80^ Mechanical parameters allow a further understanding of how chemical treatments act, and of how mechanical loading or nutrient stress affects biological differentiation. In the case of small deformations, the molecules within the biofilm come back in their original position when the solicitation is removed. The mechanical behavior of a bacterial biofilm experiencing low loadings can be described within the framework of elasticity. The deformations are so small that the deviation from the original geometry is imperceptible, and the small errors introduced by ignoring the deformation do not warrant complicating the mathematical problem. Elastic materials have the ability to absorb strain energy. Hence a biofilm can withstand transient stress events by reversible deformation. The stiffness of a biofilm influences its behavior under a stress such as flow in channels or an air-jet on teeth.^81^ The study of strength and stiffness can explain phenomena such as rippling or streaming behavior of biofilms under shear stress. To characterize the mechanical response of a biofilm subjected to low external load, the experimentalist can calculate various parameters, depending on the magnitude, the direction and the frequency of the load. In the case of an axial force, the Young’s modulus (often written *E*) can be calculated as a stiffness indicator. For a transverse solicitation (parallel to the surface), the shear modulus (often written *G*) corresponds to the stiffness indicator. Nevertheless, biofilms may be deformed even more, may ripple or flow across the surface. Moreover, biofilms can also feature time-dependent properties like viscoelastic materials. Indeed, it is known that biofilms behave like viscoelastic materials^82,83^ for larger disturbances, until a yield point from which, biofilms behave like a highly viscous liquid.^83^ This means that, depending on how fast the load is applied or removed from the biofilm, the latter is able to either store or dissipate the potential energy supplied by the load. Entanglement between polymer molecules partially governs the mechanical behavior of biofilms. For small perturbations, physical repartition of entangled molecules reorganizes temporarily but the link between the molecules acts as a permanent joint. For greater perturbations, molecules are able to slide and the global behavior of biofilm becomes more viscous.^84^ Energy dissipation is an important property that most biofilms possess, because it lets them adapt to high forces instead of being destroyed. The use of the linear elasticity assumptions would be an oversimplification and elastic moduli evoked before are no longer valid. In this case, the viscoelasticity framework is preferably used. Viscoelasticity models are identified with rheological tests such as creep recovery test or relaxation test. In a relaxation test, the sample is rapidly strained to a fixed length and the stress is recorded as a function of time. Creep recovery tests are conducted by imposing a constant stress and measuring the deformation through the time. Some rheological models are classically used to fit the viscoelastic behavior such as the Burger’s model,^81^ the Kelvin-Voigt model^85^ or the generalized Maxwell model.^50^ These latter allow to identified parameters such as apparent elastic moduli (often written *E*_*i*_) or apparent viscosity (often written *η*_*i*_), for traction, compression or shear loadings. Elastic moduli are time-dependent. Another type of test used to measure the moduli is the Dynamical Mechanical Analysis (DMA). The sample is solicited by a sinusoidal oscillating stress. Parameters inferred from these tests are called storage modulus (often written E′) and loss modulus (often written E″). Elastic, viscoelastic and plastic parameters have often been studied in the literature to determine behavior before failure under load, or the dissipative effects of the biofilm behavior. Viscoelastic modulus has already been measured under shear stress.^35,68,81,86^ Static tests report values of thickness,^87^ elastic parameters^83,88^ and tensile strength until mechanical failure.^89^

### Characterizing biofilm failure

The EPS matrix largely contributes to the biofilm together and the biofilm failure is related to the EPS loss of integrity. EPS matrix is made of many components among which polysaccharides, proteins, nucleic acid or lipids. The concentration of polysaccharides is generally high and polymer chains are therefore entangled. The composition of EPS matrix changes according to bacteria strain, growth conditions, and nutriment availability. Through the life cycle of biofilm, physico-chemical binding forces^90^ between these components manage the biofilm formation. At early stage, the contact of planktonic bacteria with the substratum involves electrostatic and Van der Waals forces. Then bacteria enhance the adhesion with ionic attractive forces or hydrogen bonds.^19^ Biofilm failure management consists in overcoming or controlling these internal forces. When dealing with biofilm failure, these physico-chemical force have to be overcome. Cell-to-surface adherence is measured to control tolerance to shear, and so the potential of biofilm survival under mechanical solicitation. Adhesion characteristics include adhesive pressure, which measures the applied stress needed to induce the removal of biofilm fragments from the substratum. Adhesive capacities of a biofilm can be inferred from tests in hydrodynamic conditions to simulate microirrigation conditions,^66,91,92^ or with directly imposed forces.^93,94^ Under hydrodynamic conditions, the biofilm undergoes a pressure due to the fluid, and the percentage reduction of the biomass or of the surface coverage is measured. Another relevant physical property is biofilm stickiness, a measure of the retraction capacity between a biofilm and a surface after a brief contact. Adhesive strength depends closely on surface properties: roughness, wettability, surface energy, and hydrophobicity of the substratum.^95,96^ Biofilm detachment is a natural phase in the biofilm cycle.^22,61^ The biofilm life cycle includes a phase of active dispersal, triggered by various signals such as starvation, which allows the cycle to continue. However, biofilm dispersal is also due to passive dispersal by sloughing,^97^ erosion or rippling in the case of shear stress. Biofilm bacteria can move in numerous ways: collectively, by rippling, rolling across the surface, or by separating in clumps, and individually, through a “swarming and seeding” dispersal. Two modes of detachment have so far been observed. Erosion is a surface phenomenon: it can be summarized as a detachment of a few cells or small aggregates at the top of the biofilm, at the fluid/surface interface. Sloughing is a more significant phenomenon in terms of quantities of detachment: sloughing is the term used to define the detachment of large pieces of biofilm.^98^ The link between the location of a decrease in bacteria density and the detachment of large pieces of biofilm has already been studied. In both cases, the detachment of biofilm results from a loss of integrity of the matrix, also called failure. Cohesive failure occurs when parts of the biofilm separate, while some others remain attached to the substratum. When the biofilm detaches from its base at the biofilm/surface interface, the failure is referred to as adhesive failure. Cohesive failure occurs when load exceeds ultimate strength of the biofilm itself, adhesive failure when stress is greater than adhesive resistance. These latter two are expressed as the ratio of a force and a surface area, or an energy. These are the relevant parameters to use when studying the conditions under which biofilm failure occurs. The relevant variables are limit shear stress,^92,99^ adhesion strength,^91,100–102^ and cohesive energy.^103^ The overall aim is to find the right conditions under which the biofilm can be actually destroyed.^104^ Moreover, viscoelasticity has to be studied to define the resistance to biofilms destruction by mechanical forces. Actually, the viscoelasticity of biofilms participates to their recalcitrance.

## Challenges in biofilm mechanics

A biofilm is not a straightforward material: it is a complex, highly heterogeneous living medium, and so is constantly evolving. Such properties yield high variability in results that have to be taken into account. This section draws attention to the high variability in the mechanical responses of biofilms and the difficulty to have repeatable results, which are among the main obstacles when dealing with the standardization of the biofilm mechanical characterization. The measurement of mechanical properties needs to reach an agreement in the community in order to validate protocols such as screening of molecules, cleaning procedures or adjusting operational parameters in industry impacted by biofilms presence.

### A biofilm is a living material

Because a biofilm is a living structure, its shape and mechanical characteristics change over time. Biofilm structure and mechanical properties depend strongly on the growing environment.^105,106^ The temperature, surface energy and hydrophobicity of the substratum,^95^ pH,^32^ flow rate in fluid conditions,^107^ and nutrient and oxygen availability are some parameters liable to affect the differentiation of bacteria and biofilm formation. Changes in the growth conditions may impact the physical properties of a biofilm, which in turn are linked to its mechanical properties.^108^ Overall, the growth conditions act on the biological and physical behavior of bacteria according to their survival potential. Bacterial specificity can change during the life of a biofilm due to the properties of bacteria as living organisms. This is why, it is nearly impossible to have a standardized sample shape like a test specimen of an engineering material and inter- testing method variability are expected. Sample configuration and experiment conditions should affect the result of the mechanical test and the comparison between two experiments. Biofilms are fragile living tissues, and are liable to be reshaped in the course of handling. This raises the possibility that the biofilm self-adapts under a mechanical load, and alters its mechanical properties to persist. Moreover, reported plastic deformations,^109^ i.e., permanent deformations, emphasize the need to grow biofilm in situ. The sample is thus not damaged before testing. In vitro studies are often used for greater convenience and minimal changes in biofilm matter. However, in vitro biofilms differ from in vivo biofilms^110^ in their structure. The results of in vitro tests are only an approximation of the reality for which assumptions have to be clarified. Moreover, due to the viscoelasticity of biofilms, flow conditions may infer modification in biofilm shape. In irrigation conditions, biofilm can roll or turn into streamers.^39,40^ In that case, the experimentalist has to adapt the way he identifies the mechanical properties in terms of force applied on the biofilm and deformation of the sample. The living property of biofilm forms a significant hurdle towards the standardization of mechanical tests. It challenges the inter-experience comparison and the accuracy of the mechanical identification.

### A biofilm is a heterogeneous material

**Fig. 1:**
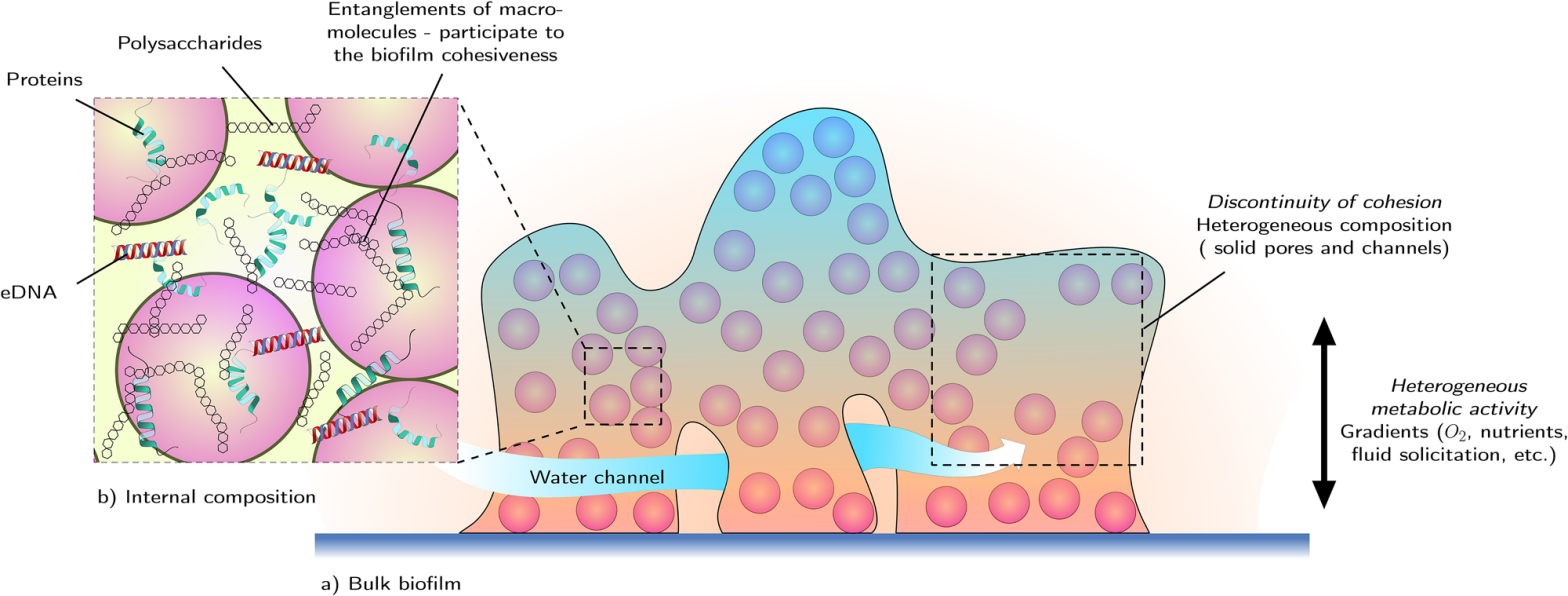
Physical heterogeneities of biofilms: biofilms are heterogeneous in their composition. **a** Biofilms are made of reinforcements (bacteria) surrounded with matrix (EPS). The influence of the scale of the mechanical study is not insignificant. Moreover, metabolic gradients (oxygen, nutrient, physical stress, etc.) result in heterogeneity in mechanical parameters. **b** A focus on the internal composition reveals that EPS matrix is made of many components. Entanglements of molecules within the EPS matrix have a key role in the biofilm behavior

A biofilm is a multiscale composite material. Depending on the point of view, the biofilm material can be seen as an homogeneous entity or as an highly heterogeneous and complex material formed of cells embedded in an extracellular matrix. As composite materials, biofilms are made of reinforcements, here the bacteria, and of a matrix, here the EPS. Moreover, biofilms is a porous matter since the EPS matrix is crossed by pores and channels through which fluid charged with nutriments can move. Fig. 1 represents the complex structure of a biofilm. Structural heterogeneity of biofilm is increased by permeability, nutrients and oxygen gradients.^111^ These gradients result in shifts in metabolic activity and impact EPS production. This stratification is accompanied by corresponding physical properties. The heterogeneity of its mechanical properties is directly related to the local microstructure of the biofilm. There is a correlation between microscale morphology, macroscale configuration and mechanical behavior of a biofilm. For example, the Young’s modulus evolves with microcolony size and biofilm shape^112^ in *P.aeruginosa* biofilms. This phenomenon is due to the increase in polymer secretion at later stages of development. The Young’s modulus of the matrix polymers influences the way the biofilm develops. The mechanical parameters differ along the directions of the biofilm sample.^80^ Beyond the heterogeneity of the EPS matrix, the variation of cell density within the biofilm infers particular mechanical behavior of the biofilm. Homogenization techniques have been used^113^ to study the influence of the biofilm spatial structure on the mechanical parameters. Homogenization techniques showed that the stiffness of a biofilm is linked to the density of the bacteria embedded in the matrix. They also proved that the detachment phenomenon in a biofilm obtained with wall shear stress is directly correlated with the heterogeneities in stiffness within the biofilm. The volume fraction of cells embedded in the EPS matrix is then a significant factor in the biofilm integrity. Actually, the bacteria within the EPS matrix constitutes solid pores which can influence the matrix strength according to the fraction of cells. Moreover, the water content in the biofilm impacts the physical properties of the matter.^114^

The biofilm characteristics discussed above make mechanical study challenging. Most experimentalists consider biofilm to be a homogeneous material, and so analyze mechanical responses averaged over the whole sample material. However, almost all biofilm characteristic properties such as biomass, concentration of chemicals or gene expression, measured at the micron scale, have been shown to exhibit strongly heterogeneous spatial distributions. Averaging biofilm at the macroscale smooths out its heterogeneity. Bulk parameters may be significantly different than microscale parameters.^102^ Thus the choice of the scale of the mechanical study is important.

### Implications in mechanical characterization

Any practitioner in biofilm mechanics has to be aware of the variability in the results of the measurements. Different setup lead to different result. Actually, medium used, planktonic model, flow situation, temperature and sampling point are various parameters which can vary the results. Moreover, in mechanics of materials, a test sample is assumed to be homogeneous or structurally determined, although a test result is always disturbed by noise during measurement, and so there is some variance in the results. By contrast, in biofilm mechanics, the sample is always different between two successive tests because of the inherent variability of the biofilm: in addition to the variance of the measurements, there is thus a further variability in the values which are reported. In the literature, deviations among reported values for the mechanical parameters may be due in part to differences in sample origin: bacterial EPS production or physical and chemical origin. However, it is likely that the difficulties met in conducting any mechanical study of biofilms include non-biological sources of variation. The complexity of this material makes any mechanical study of biofilms challenging, and identifying mechanical parameters especially difficult. This following section aims to call the reader’s attention to the intricacies of biofilm study, when several microbiological targets need reliable quantitative indicators to draw up and compare protocols for improved control of biofilms.

## Mechanical tests are abundant

Lack of standardization results in a great diversity of biofilm tests. The practitioner wanting to test a biofilm sample is faced with a bewildering array of available tests. Furthermore, methods for identifying parameters are not always clear. For classic engineering materials such as steel or concrete, testing devices and methods for the identification of mechanical parameters are largely known, and machines for standardized tests are available in most laboratories. It is tempting to transpose to biofilms, techniques that are applied to classical engineering materials, but the complexity of biofilm structure means that the conventional mechanical test benches used in industrial engineering is unsuitable. Most mechanical testing machines are sized to develop forces up to thousands of Newtons. Moreover, it is nearly impossible to secure a biofilm sample with grips for a tensile strength test and extensiometric sensors are inefficient for the measurement of biofilm deformation. The experimental determination of mechanical properties is thus a quite demanding task for materials like biofilms. In addition, the properties that describe the mechanical capacities of a biofilm are remarkably numerous and varied. Their identification is a further challenge, in view of the diversity of testing methods that take various approaches and report different results.

### Dedicated mechanical devices

**Fig. 2:**
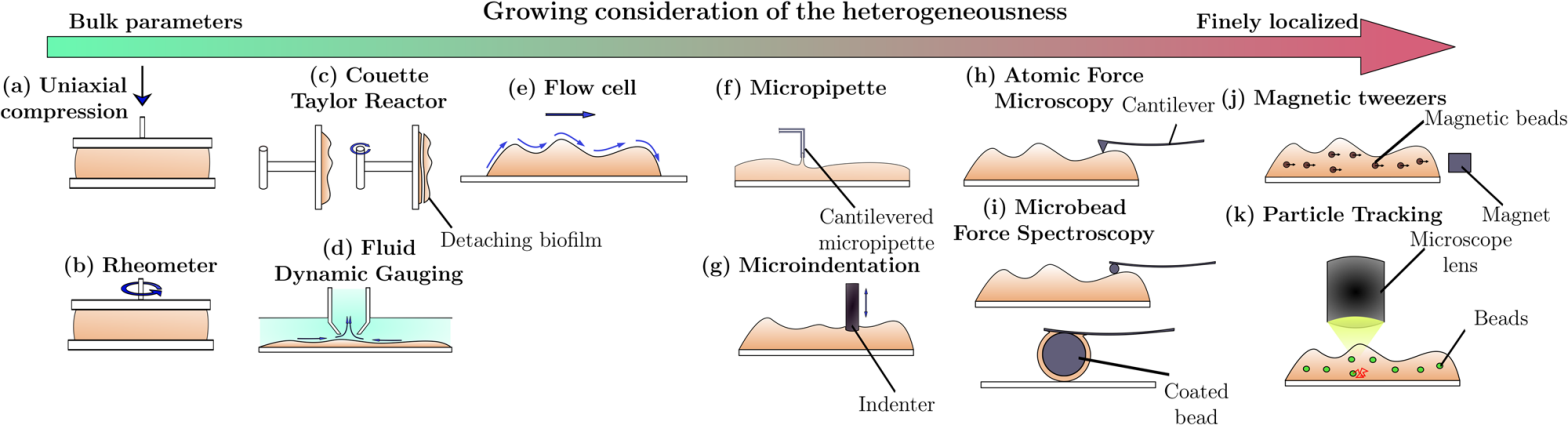
Illustrations of the most popular mechanical tests on biofilms available in the literature. **a** Uniaxial compression test; **b** Shear stress in a rheometer; **c** Hydrodynamic shear stress imposed with a Couette-Taylor reactor; **d** Fluid dynamic gauging; **e** Microirrigation condition in a flow cell; **f** Microscale tensile test with a micropipette cantilever; **g** Microscale indentation test; **h** Atomic force spectroscopy—nanoindentation; **i** Microbead force spectroscopy; **j** Microrheology with magnetic tweezers; **k** Particle tracking

The difficulty of any mechanical study lies in best matching the model with the real behavior of the biofilm, and identifying its parameters. This generally relies on reverse approaches, i.e., the determination of causes from effects. In practical terms, we measure the stationary or the time-dependent response to an imposed load or displacement. The process of identification then requires experimental data obtained from mechanical testing. Identification and testing steps are thus highly interdependent. Characterizing the mechanical properties of a biofilm relies then on the consistent combination of practical devices, microscopic techniques, and analytical methods. There is no method that will identify all the mechanical characteristics of a biofilm with a single test. Each method provides one or two parameters, based on a set of mechanical assumptions. The large number of methods emerged from several scientific groups attests to the complexity of the mechanical study of biofilms. The choice of the mechanical test and measurement device depends on the type of biofilm (biofilms on a solid surface or at the air-liquid interface), the spatial scale of the parameter involved (cf. section “Measuring relevant mechanical parameters”), the identification model and the range of the load or of the corresponding parameters. Knowing how to choose the most relevant technique to obtain the data needed requires specialist insight, knowledge, and experience. The literature seldom specifies a domain of validity for each method of characterizing biofilms. The techniques used for their characterization have been fully described in previous work.^13–15^ They are based on rheology, microrheology or engineering test. They are briefly reviewed in what follows. The main criteria for the validity of a standard method are reproducibility, repeatability, relevance, and ruggedness. Two main strategies for loading and measurement are outlined: the measurement of macroscopic properties for the whole tested biofilm, and the calculation of microscopic properties within the biofilm. The methods are illustrated in Fig. 2. Their domains of applicability will be fully exposed to the reader in the following guideline section “Guidelines for studying mechanics”.

### Biofilm considered as a homogeneous material

Despite the heterogeneity of biofilms, bulk parameter testing devices are still often used to characterize their macroscopic mechanical properties. Bulk rheology offers the advantage of being easier, but it requires making significant simplifications to the mechanical parameters. The best-known tool for the rheology of viscoelastic materials is the rheometer. It is still largely used because of its ease of application and its availability in most laboratories.^102,115^ It is also possible to cultivate in situ biofilms on the rheometer disks in a growth reactor under a rotational fluid shear stress, and so biofilms samples are not scraped. Moreover, the rheometer allows to conduct dynamic tests thanks to its oscillatory mode.^116^ One of the limitations of this device is the need for a large amount of biofilm to cover the rheometer plate. Another drawback of this method is the non-constant shear rate across the radius of the sample, which complicates the mechanical identification. It is worth noting that, usual rheometers can also be used to perform uniaxial compressive tests. Stress/strain curves are derived from the measurements of biofilm depth and axial force exerted by the upper plate.^83^ The main remaining difficulty is the biofilm sample bulging. Compression testing has also been implemented between two glass rods.^117^ Low load compression testing (LLCT) has been more recently developed for uniaxial compressive tests. The difference is the 2.5 mm diameter cylinder upper plate that bears down on the biofilm sample with up to 20% strain.^87,107^ The same properties as for compressive tests are then inferred. Other custom-built devices have emerged since the early 1990s but have been used with more sparsity. Centrifugation^91^ technique have been used to measure cohesive shear stress. Biofilm-bearing plates are placed on a rotary table, perpendicularly to the radius of rotation. The biofilm experiences a body force acting in the plate’s normal direction that pulls it away from the adhesion area. This leads to the detachment of the biofilm when the adhesion strength is overcome. This corresponds to the stress before the detachment of the biofilm. However, the output data are not easy to interpret. Much depends on the intrinsic parameters of the centrifuge machine such as the discretization of the centripetal acceleration. Moreover, extraneous effects can occur: gravitational force may cause vertical deformation during the fixation of the sample on the rotary table; continuous acceleration may cause gradual detachment of different masses; the centrifugation results in residual inertial force causing shear stress that may contribute to premature detachment of the biofilm. For tensile strength tests at macroscopic scale, biofilm grows at the external surface of a pair of adjacent tubes.^118^ One of the tubes is loaded axially so that the tubes are pulled apart until they separate. Using a camera, measurement can be made to determine detachment and plot a stress/strain curve. For tensile test on pellicle-shaped biofilm,^109^ the device is reconsidered as the tension is applied in the horizontal plan, at the air liquid interface.^119^ Flow-cell remain a largely used technique for the study of cohesive shear strength or of the viscoelastic behavior of biofilm in irrigation conditions. In flow shear stress experiments, a biofilm grows in a microscale channel under a laminar flow. To test the biofilm, the flow shear rate is modified and the displacements of biofilm clusters are imaged with a camera. The flow cell is a versatile and relatively cheap technique which allows a precise manipulation of the fluid flow. Most laboratories already use this well-known method. These main practical drawbacks are the uncertain knowledge of the force applied to the biofilm surface and the need of numerical integration. Basically, the main source of problems in mechanical measurement is the uncertain value of the stress directly applied to the biofilm. More recently, fluid dynamic gauging has been implemented for testing biofilms in microirrigation conditions to circumvent this. This uses a biofilm-non-contact device: a gauge nozzle of known diameter is immersed in a liquid and placed under a thin biofilm, at a set distance. A pressure drop is imposed across the nozzle, causing flow of liquid through it. This technique determines thickness, cohesive and adhesive strength of the biofilm in resistance to shear stress.^66,120,121^ The shear stress imposed on the biofilm depends on the pressure drop, the nozzle-to-biofilm distance, and the nozzle diameter^122^ and is thus known.

### Considering biofilm heterogeneity

As discussed previously, biofilm mechanical properties present spatial heterogeneities due to the biofilm structural organization. Since the beginnings of biofilm observation, the development of technologies such as confocal laser scanning microscopy (CLSM)^123^ have allowed the tracking of biofilm deformation under loads at smaller length scales. The testing devices developed in the community for the study of biofilm sometimes derive from living cell mechanics or from classical rheology. At the microscale, uniaxial compression tests are possible with microindentation devices. The microindentation device is a testing machine that applies a known force at the surface of a biofilm grown on a glass surface with an indenter. In microscopic compression, a microindenter of known geometry (conical, spherical, flat plate), and of small dimensions compared with the sample to be characterized is used. The indenter compresses the material locally and gradually. A curve of applied load versus indenter depth is experimentally plotted during a cycle of loads and during an unload. The displacement of the tip is recorded and a stress/strain displacement is plotted. This method determines elastic parameters during compression experiments or viscoelastic characteristics in relaxation tests. The advantage of microindentation is the possibility it offers of determining mechanical properties at a small scale.^65^ One of its main drawbacks is that compression is not the natural force a biofilm is most likely to undergo. For several years atomic force microscopy (AFM) has been largely used for the characterization of viscoelastic parameters and adhesive properties. This technique is feasible in static or dynamic mode. AFM measures adhesion and cohesion force.^93,103^ AFM is also a common technique used for imaging surfaces, and probing the 3D mapping of surface roughness. For the study of adhesion, diverse modified tips have been implemented in AFM, such as coating or probe tips. The range of applied forces is wide: from piconewtons to several nanonewtons. Coated tips have been used to characterize adhesive properties and viscoelastic characteristics.^93^ Thanks to its simplicity which signifies here reliability and easiness of implementation, numerous mechanical testing devices rely on cantilever. For instance, a microcantilever is used to test biofilms with tensile stress.^89^ The biofilm sample is pipetted with one or two pipettes. One pipette serves as a microcantilever. The tensile test is recorded with a video capture device to obtain the deformation of the biofilm part. Stress is deduced from the deflection of the cantilever from its initial position and its bending stiffness. Adhesive profiles have recently been investigated with the retraction curves based on a microscale AFM method.^117^ Some research groups have designed custom-built devices for the characterization of biofilm properties. The T-shaped probe^94^ is a micromanipulation technique used to study adhesive strength. The work per unit of area needed to remove the biofilm with the T-shaped probe is measured. More recently, several microrheology techniques have been adapted to the study of biofilms.^80,124,125^ Optical tweezers use a laser beam focused on a polarizable particle. Magnetic tweezers are paramagnetic beads embedded in the biofilm, which are moved with a permanent magnet or an electromagnet.^80^ These techniques give access to the microscopic, localized properties of biofilms. They use microscopic probes embedded in the biofilm medium. In the same way, passive microrheology consists in recording the movements of inert beads embedded in the biofilm. No forces are applied on the beads: their displacement is thus due only to the thermal fluctuations. To determine the elastic properties, the particle motion is assumed to be driven solely by thermal fluctuations. However, the activity of motor proteins and other non-equilibrium processes in cells also contribute to motion. Omitting to consider these effects can produce erroneous results.^126^

## Identifying mechanical parameters: a source of confusion

**Fig. 3:**
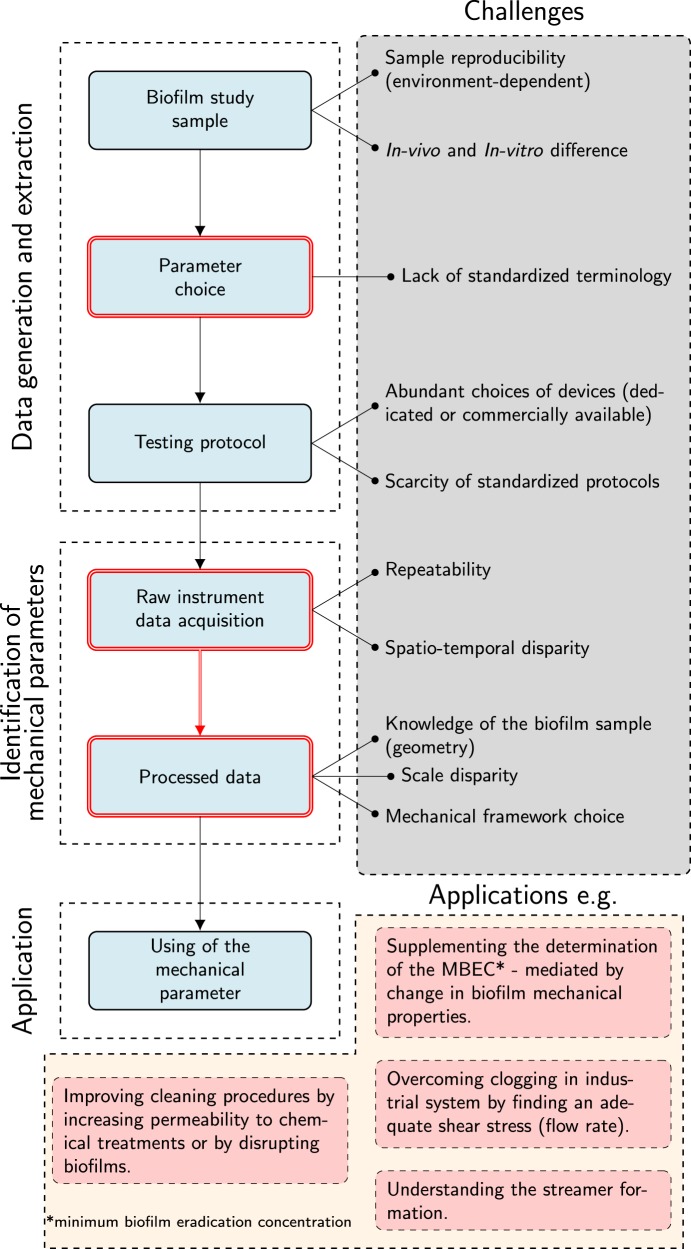
Global life cycle of biofilm mechanical studies: due to the complex structure and living properties of biofilms, challenges exist in each step of the identification of mechanical parameters. The red double lines indicate the points that are addressed in this manuscript. Overcoming these challenges is appealing as the applications of the mechanical properties must be of particular interest

In mechanics, identification means finding the values of constitutive model parameters from experimental data. The experimental measurements made with each of the above devices provide raw data. The processing of these data and the matching of mechanical forces with resulting transformation are used to infer mechanical parameters. It transpires that several methods can lead to the same parameters because they target the same mechanical phenomenon. The difficulty is to be able to choose a combination of test device and identification method that is right for both the sample and the parameters concerned. Besides the choices of mechanical test device, the identification method needs to be thought out in depth. Overall, the choice of the identification method must be made according to the mechanical parameter at stake. Likewise, the testing method should emphasize the mechanical behavior consistent with the aforementioned mechanical parameter. Fig. 3 shows the global methodology for the mechanical identification of parameters in biofilms study and reveals challenges that currently hampered the standardization.

### Misleading about identification

Because of the special features of biofilm matter and the diversity of available methods to test biofilms, the identification step can be a source of major discrepancies. Owing to the variety of experimental conditions reported in the literature, caution should be exercised in interpreting data resulting from a mechanical study. As discussed above, variability is expected in the results of the same test on two samples of a biofilm grown in the same conditions. Due to these difficulties, the literature contains some misleading links between experimental data and identification methods. Most confusion occurs in the choice of the definition of elastic moduli or the choice of the behavior laws. The various ways to identify parameters might lead to different interpretations of the test and sometimes would infer unreliable results if wrong assumptions are made. In the literature, the question of whether an identification method is relevant to a particular problem is seldom addressed. A common mistake is to associate any stress/strain ratio to Young’s modulus, even when the material does not behave linearly or when the geometry of the sample and the applied load is inappropriate. Young’s modulus is the ratio of axial stress to axial strain. Under no circumstances must Young’s modulus be calculated as the ratio of shear stress to longitudinal deformation. Moreover, many research confuse elastic and linear. Stress/strain curves and force/displacement curves are strongly interdependent in the simple case of geometric and material linearity and for axial loading. In this case, stiffness is a linear characteristic. In other cases, depending on the material behavior, loading and non linearity will affect the stress/strain curve. Nonlinear elasticity is an other existing type of behavior. In classical engineering, loadings are usually well known, in terms of direction and area of application. In the case of biofilms, surface areas under load are often imperfectly known because of the roughness of the biofilm surface or their non-regular shape. Moreover, biasing means are sometimes hydrodynamic or indentation within the matter and thus unchecked precisely. The reader has to be mindful of the homogeneity of the stress applied before using formulas. After reporting mistaken identification of Young modulus in a previous study, a research group presented a methodology^127^ for the calculation of Young’s modulus of biofilm streamers. The method, based on a correct calculation of elastic parameters, explains the deviations of one order of magnitude between two results. This is the case in the linear elastic theory. Finally, the reader has to make a choice in terms of measurement resolution. We can consider a biofilm as a heterogeneous material whose properties depend on the scale at which they are calculated. Two main scales of exploration of biofilm properties are possible and relevant depending on the target of the mechanical study. Microscale parameters can be relevant for the study of biofilm metabolism and physical organization within the biofilm. Mesoscale or macroscale studies allow the determination of the influence of fluid/biofilm surface interaction or stress tolerance whereas emerging imaging techniques can be used to study biofilms with microscale resolution. Since biofilm structure is complex, biofilm properties change markedly from one point to another. Depending on the scale of the measurements, results may be different. Homogenization techniques have been applied to quantify macroscopic mechanical properties of heterogeneous materials such as bacterial biofilms.^113^

### Full-field measurement methods

The heterogeneity of biofilms is one of the main challenges when we are studying mechanical behavior. We have seen that mechanical devices allow microscale parameters to be determined. However, some testing techniques probe microscale parameters in a localized part of the biofilm, whereas biofilms often exhibit different mechanical properties depending on location. An emerging tool to access the real behavior of the sample under loading is the use of full-field measurements methods. Full-field measurement techniques provide full maps of mechanical quantities of interest such as strain map over a specific biofilm region. These methods are useful when we cannot use a sensor. They are non-invasive, an important feature given that we know biofilms can be disrupted by any modifications to their environment. Finally such methods provides maps instead of localized measurements. Particle image velocimetry (PIV) and Digital image correlation (DIC) are still largely studied and used in the field of fluids and solid mechanics respectively. PIV has been used to map the displacement on pellicles loaded with axial tension.^109^ DIC has been implemented on biofilm clusters to measure the displacement of biofilms in flow cells under wall shear stress.^128^ Both PIV (resp. DIC) relies on images of the material of interest before and during testing to determine velocity (resp. displacement) maps. Recently, another non-contact method, the Brillouin microscopy, has been implemented to probe the internal stiffness of biofilms colonies.^129^ The Brioullin microscopy measures a frequency shift of light incident upon the biofilm sample. The frequency shift is then correlated to an evolution of stiffness of the matter. Full-field measurements are promising identification methods especially since increasingly well-controlled image techniques and tools are becoming available. Confocal scanning laser microscpy (CSLM) is also now largely used^130^ and Optical coherence tomography (OCT)^131^ is being developed and beginning to be used for biofilm studies.^132^ OCT allows fast imaging up to real-time acquisition, is resistant to more extreme conditions of testing, and is faster than other imaging techniques. Full-field measurements methods are also consistent with the idea of “biofilm-friendly” growth conditions. They allow real-time acquisition, and hence easy addition of a temporal character to the mechanical test. Finally, full-field measurements are able to obtain in vivo, dynamic and multiscale information.

## Guidelines for studying mechanics

**Fig. 4:**
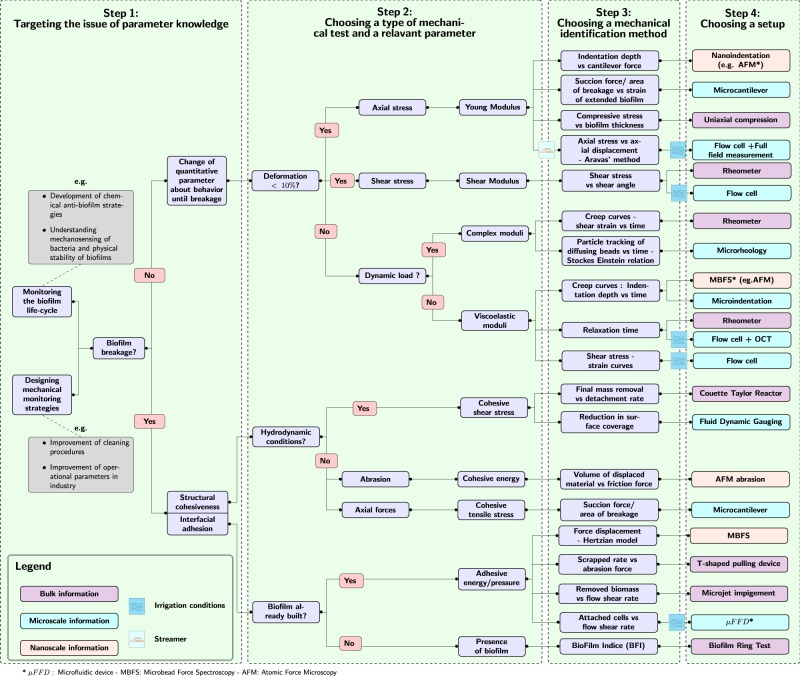
Guidelines for helping the choice of mechanical parameter, testing method and identification method. From left to right, the reader is invited to follow the different steps of the identification of mechanical parameters. In the first step, the microbiological target is determined. Then, a relevant mechanical parameter is advocated according to the conditions of solicitation of the biofilm sample. Final steps 3 and 4 suggest the reader, a choice for a mechanical setup and an identification method, which are relevant with its microbiological issues. Step 3 and 4 can be reversed depending on the circumstances

The following part sets out some rules of good practices to avoid pitfalls and respond efficaciously to the challenges of biofilm study. The following reasoning is depicted in the guideline in Fig. 4. On the basis of the microbiological issues, the reader is invited to question on several features according to the relevance of the mode of solicitation of its biofilm sample. Four steps have been determined for the proper conduct of the mechanical study of a microbiological issue. In our opinion, this guideline is a necessary clarification to lay the groundwork towards potential standardization.

### Step 1: Targeting the issue of parameter knowledge

The degree of relevance of the use of mechanical parameters depends on the microbiological issue (cf. “Biofilms mechanics: a microbiological significance”). Thus the characterization of the mechanical properties of a biofilm must begin with a clear definition of the microbiologist’s objectives. For research into the physiological or morphological mechanisms operating inside biofilms and especially between EPS molecules, the experimentalist should probe a change of a quantitative mechanical indicator before the loss of biofilm integrity. In the case of the antimicrobial screening of novel drugs or the development of mechanical removal strategies, evaluating an accurate threshold value until which the biofilm remains undisturbed is more valuable for the direct application in industrial context. The properties of every material are defined relatively to a mechanical state of the biofilm, and different issues require different mechanical aspects to be considered. Some parameters are related only to low deformation, without biofilm failure, whereas others characterize the detachment or breakup of the biofilm. This latter can be viewed in terms of (i) cohesive failure, when bulk parts of the biofilm detach whereas the rest of the biofilm remains attached, or (ii) adhesive failure, when all the biofilm losses its adhesion on the substratum.

### Step 2: Choosing a type of mechanical test and a relevant parameter

It is necessary to know at the outset what kind of behavior a biofilm sample displays. If a biofilm is expected to have an elastic behavior, a static test may be done. Three types of loading lead to relatively well-known stress states: tension, compression, and shear loads. Elastic behavior is consistent for small deformations only. Care must be taken to ensure that the displacement generated does not entail geometrical non-linearities (i.e., large strains). Strains of more than 10% shall be considered as large strains. If a pre-test shows a time-dependent relation between applied stress and resulting displacement, then the biofilm shall be treated as a viscoelastic material. In that case, two types of tests can be performed. Transient tests include the creep test, in which a constant stress is applied and the strain is measured, and the relaxation test, in which the decrease in stress is measured under constant strain application (creep compliance). Spring-dashpot models are then used to determinate the viscoelastic modulus. For viscoelastic behavior, it is also possible to carry out dynamic tests, consisting in applying oscillating stress or strain with a known frequency. The phase angle on the strain or stress amplitude is then measured. If the behavior is viscoelastic, then the response to oscillating stress will be sinusoidal strain. The complex viscoelastic modulus *G* = *G*′ + *iG*″ is determined with an oscillating loading, where the storage modulus *G*′ represents the elastic part of the mechanical response whereas the loss modulus *G*″ reflects the damping capacity of the biofilm sample. Once the parameter of interest is determined, the scale of the sample has to be chosen. The type of test determines the scale of the objective mechanical parameters. Depending on the situation, the importance of having microscopic values will vary. For example, designing a device for removing a biofilm with a water flow may need only average parameter values, whereas the study of physiological mechanisms within the biofilms will need the description of local properties, at least at the bacterial scale. It must be taken into account that the timescale of the mechanical test is also of particular importance as biofilm is a living matter which constantly evolves.

### Step 3: First determine which identification method is suitable

The mechanical test is going to give yield data that we need to process to infer mechanical properties according to theoretical models of behavior. Most of the existing mechanical tests measure raw data, force, and displacement, for the test sample. In order to have general parameters, which do not depend on the particular sample but on the bacterial strains and the growth conditions, these values are converted into specific magnitudes (force per unit area and displacement per unit length). This step can sometimes be misleading. The complex geometries of the biofilm specimens with the limited two-dimensional view provided by the microscope require some simplifying assumptions that may introduce error into the estimates of the load (i.e., stress) applied to the specimen during testing. The choice of the identification is a significant step, because this is the main source of error, particularly regarding assumptions made and definitions. The identification of mechanical parameters is based on the hypothesis of simplified models. Resulting mechanical parameters depend directly on the assumptions about models for the shape of the biofilm and its constitutive law. The geometry of the sample is thus an important parameter in any mechanical study: the transfer of the external load to the biofilm medium depends closely on the area of contact. The experimentalist has to know the shape of the sample to investigate its mechanical properties meaningfully. We assume the material behavior of the biofilm is shown in the test curve profiles. Biofilms are known to be viscoelastic materials. In the case of small strains, biofilms can have elastic behavior. A force must be chosen that keeps the test sample in the linear elasticity domain. The Young’s Modulus can be used only if a material has, even partly, a linear elastic behavior. In the simple case of elastic linearity we can determine stress/strain curves from force and displacement by dividing the applied load by the cross section, assuming the stress is constant throughout the sample. The slope of a stress/strain curve must not be unthinkingly equated to Young’s modulus: there is a risk of distorting the physical identity of this property. An identification method can be selected according to the mechanical assumptions and the particular configuration of the biofilm to be tested. One test can be used to infer different parameters, but the identifying method can be different.

### Step 4: Then choose a setup

Once we know what kind of experiment we are going to do, we have to choose a mechanical device to implement it. Mechanical tests described in the literature allow the prescribing and the measurement of different quantities. The choice of the test method will depend on several parameters such as the microbiological objectives, the biofilm state of maturation (a young biofilm can hardly be tested with attached tubes), the type of biofilm (bacterial or yeast biofilms, pellicle or surface-attached, etc.). Several methods can lead to the same parameters because they target the same phenomenon, but the optimal method for identification could be different. In any case, the duration of the test must be short in order to avoid evaporation of the hydrated part of the biofilm, and to be free of changes in the properties of the biofilm due to its living status. Each mechanical test has to be repeated to average the results. Step 4 and aforementioned step 3 are exchangeable, as appropriate.

## Discussion and concluding remarks

Issues addressed in the article are discussed in the form of three main messages:

### Mechanical properties are unfortunately equivocal

Although biofilm mechanisms are now increasingly well understood, there is still a need to study the mechanical response of biofilms. Mechanical parameters should be useful for better understanding the mechanisms within the biofilm throughout its life-cycle, for developing cleaning strategies by determining necessary mechanical forces of disruption, for optimizing industrial processes by finding the flow stress needed to override biofilm presence or for implementing antimicrobial screening. Examining in depth how better knowledge of mechanical properties can be taken into account to impact the aforementioned issues will justify dedicated studies. Several research topics take support from mechanical considerations to elucidate the complex mechanisms that operate within the biofilm during its growth and dispersal. However, the mechanical study of biofilms is still very challenging. We have seen here the gap between the desire to understand the material properties of bacterial biofilm and their successful mechanical characterization, which requires experimental data and their interpretation through an identification step. Because biofilms are complex, heterogeneous, living media, their mechanical study is still much more difficult than that of classic engineering materials. Dedicated mechanical devices for biofilms have accordingly been implemented. Each of these has a range of action, with advantages and drawbacks.

### A guideline for testing biofilms is essential for an interdisciplinary community

Until now, research on mechanical parameters has often been disconcerting owing to the diversity of the testing machines available and the variations, over several orders of magnitude, of the results reported in the literature. It is difficult for researchers to find earlier results to compare with current ones. The deviation between two research groups can be wide for the same mechanical features and the same growth conditions. Bias still hamper the pertinence of the analysis of the mechanical measurements such as, amongst others, the scarcity of homogenized testing protocols, the lack of normalized vocabulary, the difficulty of testing repeatability and reproducibly. Emerging platforms aim at standardizing and structuring the data sets required for the consistent publication of results. This makes easier the comparison of results obtained in different environments. Yet, the choice of a dedicated procedure for the identification of specific mechanical parameters still remains unclear and its implementation furthermore represents challenges upstream. Thus a “guide” to understand the issues and difficulties of the mechanical identification has been drawn up. Its objective is to clarify the bases of the different steps of any mechanical study of biofilms. Our purpose is for the novice to become more knowledgeable about biofilm mechanics and acquire a deeper understanding of the relevant mechanical testing. The guidelines proposed here will help the reader find a suitable testing device and obtain the data to be extracted according to the microbiological issue concerned and its attendant assumptions. This problem has seldom been addressed in the literature. Nevertheless, this does not constitute standards as the latter requires standard-setting body.

### Developing standards relies on good practices

There arises the issue of standardizing biofilm mechanical studies. A key aspect of future biofilm research is the need for standards: a unified terminology and well described protocols are required. These guidelines are a preliminary step in the direction of a potential code of good practices. They are designed as a list of recommendations with rules for the proper interpretation of experimental data. Well-established quantitative mechanical data will enable a reliable comparison of experimental mechanical parameters within and across laboratories. Moreover, it will facilitate the peer-reviewing for the validation of data. Besides the current needs in terms of management strategies, the mechanical study of biofilms is required for the numerical implementation of biofilm models and the enrichment of databases for finite element analysis. Modeling biofilms with numerical simulations is an important part of future research. Modeling new sources of biofilms is beginning to be addressed in the literature, e.g., multispecies biofilms^133^ or biofilm aggregates.^2^ Numerical simulations also lead to better insight into biofilm permeability by modeling fluid on biofilm void.^134^ The reliability of such simulations inherits from the accuracy of the implemented models and of their calibration thanks to consistent experimental testing.

### Data availability

Data sharing not applicable to this article as no datasets were generated or analysed during the current study.
